# Nuclear Translocation of SRPKs Is Associated with 5-FU and Cisplatin Sensitivity in HeLa and T24 Cells

**DOI:** 10.3390/cells10040759

**Published:** 2021-03-30

**Authors:** Ioanna Sigala, Maria Koutroumani, Anastasia Koukiali, Thomas Giannakouros, Eleni Nikolakaki

**Affiliations:** 1 Laboratory of Biochemistry, Department of Chemistry, Aristotelian University, 54124 Thessaloniki, Greece; isigala@chem.auth.gr (I.S.); akoukiali@chem.auth.gr (A.K.); giannako@chem.auth.gr (T.G.); 2 Centre for Research and Technology-Hellas, Institute of Applied Biosciences, 57001 Thessaloniki, Greece; maria.koutroumani@certh.gr

**Keywords:** SRPK1, SRPK2, SR protein kinases, 5-FU, cisplatin, drug resistance, ATM, ATR, fixation for immunofluorescence

## Abstract

Serine/arginine protein kinases (SRPKs) phosphorylate Arg/Ser dipeptide-containing proteins that play crucial roles in a broad spectrum of basic cellular processes. The existence of a large internal spacer sequence that separates the bipartite kinase catalytic core and anchors the kinases in the cytoplasm is a unique structural feature of SRPKs. Here, we report that exposure of HeLa and T24 cells to DNA damage inducers triggers the nuclear translocation of SRPK1 and SRPK2. Furthermore, we show that nuclear SRPKs did not protect from, but on the contrary, mediated the cytotoxic effects of genotoxic agents, such as 5-fluorouracil (5-FU) and cisplatin. Confirming previous data showing that the kinase activity is essential for the entry of SRPKs into the nucleus, SRPIN340, a selective SRPK1/2 inhibitor, blocked the nuclear accumulation of the kinases, thus diminishing the cytotoxic effects of the drugs. ATR/ATM-dependent phosphorylation of threonine 326 and serine 408 in the spacer domain of SRPK1 was essential for the redistribution of the kinase to the nucleus. Substitution of either of these two residues to alanine or inhibition of ATR/ATM kinase activity abolished nuclear localization of SRPK1 and conferred tolerance to 5-FU treatment. These findings suggest that SRPKs may play an important role in linking cellular signaling to DNA damage in eukaryotic cells.

## 1. Introduction

Serine/arginine protein kinases (SRPKs) constitute a subfamily of serine-threonine kinases that specifically phosphorylate serine residues residing in arginine-serine dipeptide motifs, known as RS domains [1,2]. The SRPK family is conserved in all eukaryotic cells from *Caenorhabditis elegans* to humans [1]. Originally considered to exclusively regulate pre-mRNA splicing through the phosphorylation of SR splicing factors, SRPKs are now known to be involved in various cellular processes [1,3]. As the mammalian genome contains more than a hundred RS domain-containing proteins [4], this pleiotropic mode of action might be related to the phosphorylation of diverse substrates, thereby resulting in the activation of distinct downstream signaling pathways.

Several modes of regulation of SRPK function have been described, implying an elaborate cellular control of their activity. All family members share highly conserved kinase domains, which are separated by a unique spacer sequence. The spacer region that was initially considered as dispensable for kinase activity appears to be the most important regulatory part of SRPKs. Recent evidence suggests that intramolecular disulfide bond formation within the spacer domain of SRPK1 might promote its folding into a loop-like structure, thus bringing the two catalytic domains into proximity and allowing the functional interactions between the two lobes, which is a prerequisite for the kinase to adopt an active conformation [5,6]. Furthermore, the spacer domain is the critical modulator of the cellular partitioning of SRPKs. Both SRPK1 and SRPK2 are primarily localized in the cytoplasm, while deletion of the spacer sequence forces the nuclear accumulation of SRPKs, with harmful effects ranging from the aggregation of the splicing factors and defects in the splicing machinery in mammalian cells [7,8,9] to inhibition of cell growth in yeast [10]. Interestingly, despite being quite variable in sequence, the spacer regions in SRPK1 and SRPK2 seem to function interchangeably as insertion of the spacer of SRPK2 into SRPK1 restored the cytoplasmic localization of the latter [8].

SRPKs were shown to translocate to the nucleus upon stimulation of mammalian cells by hormones or growth factors. Activation of the PI3K–Akt signaling pathway by EGF resulted in SRPK1 nuclear translocation and reprogramming of alternative splicing [11], while SRPK2 was shown to redistribute into the nucleus upon activation of the mTORC1 signaling cascade by insulin, leading to de novo lipid biogenesis [12]. On the other side, SRPKs were also classified as “stress kinases” because they mediate the cellular stress response through their translocation to the nucleus, increased phosphorylation of SR splicing factors, and alterations in the splicing machinery. Sorbitol-induced osmotic stress caused SRPK1 to increase its presence in the nucleus [9], while the entry of SRPK2 into the nucleus was observed when human neuroblastoma cells were treated with paraquat, an uncoupler of the mitochondrial electron transport chain that induces superoxide formation and oxidative stress [13]. Finally, earlier reports indicated that cell cycle signals may also trigger the translocation of SRPKs to the nucleus at the late G2 phase, presumably to facilitate cell cycle progression at the G2/M phase [8,14].

Chemotherapy can be thought of as a severe type of stress, which may then lead to cell apoptosis and death. The contribution of SRPKs to the cellular response to chemotherapeutic agents is quite controversial. Downregulation of SRPK1 expression has been coupled to both the susceptibility [15,16] and resistance [17,18,19,20] of tumor cells to platinum compounds. Surprisingly, SRPK1 expression was associated with either cisplatin sensitivity [21] or resistance [17] in the same ovarian cancer cell line, namely, SCOV3. There is also evidence that SRPK1 and SRPK2 may have different roles in response to chemotherapeutic drugs. Downregulation of SRPK2 in non-small cell lung cancer cells prevented the induction of apoptosis following cisplatin treatment, whereas downregulation of SRPK1 increased apoptosis [22]. Most, if not all, of the above studies focused on the protein levels of SRPKs as the only determinant factor in drug responsiveness. Little attention has been given to alterations in the subcellular localization of SRPKs following drug treatment. In this respect, SRPK2 relocalizes in the nucleus upon cisplatin or paraquat treatment [13,22], whereas cisplatin induces only a partial nuclear translocation of SRPK1 [22]. Interestingly, a more prominent SRPK1 staining was detected in the nuclei of cisplatin-resistant breast cancer cells [23].

Phosphorylation is the critical modification that triggers the nuclear translocation of SRPKs, yet the underlying signaling events so far described appear to be distinct. Alternatively, an acetylation/deacetylation event was also shown to regulate both SRPK1 and SRPK2 localization. Strong nuclear accumulation of SRPK1 and SRPK2 was observed in non-small-cell lung cancer cells deprived of the acetyltransferase Tip60 [22], while a GFP-tagged SRPK1 mutant, in which the predicted acetylated lysine residues were mutated to arginine, was more prone to localize in the nucleus of HeLa cells [23].

Here, we report that treatment of two widely used cancer cell lines, HeLa and T24, with DNA damage agents induced the entry of SRPKs into the nucleus, with the relocalization of the kinases paralleling the appearance of γH2AX foci. The redistribution of SRPKs from the cytoplasm to the nucleus was closely related to 5-FU and cisplatin sensitivity. Concurrent treatment of cells with 5-FU or cisplatin and SRPIN340, a selective SRPK inhibitor, diminished the cytotoxic effects of the drug. Confirming previous data that the kinase activity is essential for the entry of SRPKs into the nucleus, SRPIN340 prevented the nuclear translocation of the kinases, thus resulting in drug resistance. In an attempt to delineate the molecular mechanism underlying the relocalization of SRPK1 from the cytoplasm to the nucleus, we found that ATR/ATM-dependent phosphorylation of threonine 326 and serine 408 in the spacer region of SRPK1 was essential for nuclear translocation of the kinase. The mutation of either of these two residues to alanine or blocking the activity of ATR/ATM by pharmacological agents restricted SRPK1 to the cytoplasm. Furthermore, cells co-treated with ATR/ATM inhibitors showed increased tolerance to 5-FU treatment. These findings, coupled with previous reports that SRPK1 is associated with a large number of DNA damage-induced phosphorylation events [24], highlight the key role of SRPKs in the DNA damage response.

## 2. Materials and Methods

### 2.1. Plasmid Construction and the Expression of Recombinant Proteins

pGEX-2T-SRPK1, pGEX-2T-LBRNt(62–92) (expressing a 31-amino-acid fragment of the N-terminal domain of Lamin B receptor that contains the RS dipeptides), pFLAG-CMV-2-SRPK1, and pFLAG-CMV-2-SRPK151Α (in which Ser51 was mutated to Ala) have been previously described [24,25,26,27]. Point mutations were inserted in SRPK1 by site-directed mutagenesis using the QuickChange^®^ Lightning kit (Stratagene, La Jolla, CA, USA) following the manufacturer’s instructions. The following primers (s, sense; a, antisense) were used to mutate T326 and Ser408 to alanine or aspartic acid:

- 326 Thr→Ala (s): 5′-GAACCCACCTAATAAAATGGCCCAAGAAAAACTTGAAGAGTCAAG-3′;
- 326 Thr→Ala (a): 5′-CTTGACTCTTCAAGTTTTTCTTGGGCCATTTTATTAGGTGGGTTC-3′;
- 326 Thr→Asp (s): 5′-GAACCCACCTAATAAAATGGACCAAGAAAAACTTGAAGAGTCAAG-3′;
- 326 Thr→Asp (a): 5′-CTTGACTCTTCAAGTTTTTCTTGGTCCATTTTATTAGGTGGGTTC-3′;
- 408 Ser→Ala (s): 5′-ATGGAGACAGCAGCACAGCTCAAGAAACAGACTC-3′;
- 408 Ser→Ala (a): 5′-GAGTCTGTTTCTTGAGCTGTGCTGCTGTCTCCAT-3′;
- 408 Ser→Asp (s): 5′-ATGGAGACAGCAGCACAGATCAAGAAACAGACTC-3′;
- 408 Ser→Asp (a): 5′-GAGTCTGTTTCTTGATCTGTGCTGCTGTCTCCAT-3′.

All the mutated cDNAs were sequenced to rule out unwanted mutations. The double mutants SRPK1 326/408A and SRPK1 326/408D were generated using the single mutants SRPK1 326A and SRPK1 326D as templates for the PCR reaction.

### 2.2. Cell Culture and Transfection and Drug Treatments

HeLa, T24, M059K, and M059J cells were cultured in DMEM medium supplemented with 10% (*v*/*v*) fetal bovine serum (FBS) and antibiotics. Cell culture products were purchased from Gibco-Invitrogen, Carlsbad, CA, USA. Cells were incubated at 37 °C with 5% CO_2_. Human glioblastoma cell lines M059K and M059J cells were kindly provided by Dr. G. Iliakis (Institute of Medical Radiation Biology, Essen, Germany). Transfections of the plasmids expressing wild-type SRPK1 and derived mutants were done with the Xfect™ transfection kit (Clontech-Takara Bio, Mountain View, CA, USA), according to the manufacturer’s instructions; the cells were collected after 48 h. Briefly, 7 × 10^4^ HeLa cells were plated in 24-well plates and 1 μg of plasmid DNA was diluted with Xfect Reaction Buffer to a final volume of 50 μL and added to 0.25 mL DMEM (without FBS). Following 4 h of incubation, nanoparticle complexes were removed via aspiration and 1 mL of fresh complete growth medium was added. Cells were treated with 5-FU (5–50 μg/mL), cisplatin (5–50 µM), H_2_O_2_ (10 mM), SRPIN340 (SRPK1/2 inhibitor, 5–80 µM), KU-55,933 (ATM inhibitor, 1–15 μM), and VE-821 (ATR inhibitor, 1–15 μM), either alone or in combination, as indicated. SRPIN340 was kindly provided by M. Gammons (MRC Laboratory of Molecular Biology, University of Cambridge, UK), while all other chemicals were purchased from Sigma-Aldrich, Darmstadt, Germany.

### 2.3. Immunofluorescence Microscopy

HeLa cells were grown on glass coverslips and treated with various chemicals for the indicated periods. After the incubation period, the cell coverslips were fixed with 1%, 2%, 3%, or 4% formaldehyde in phosphate-buffered saline (PBS) pH 7.4 for 5 min or 4% paraformaldehyde in PBS for 20 min at room temperature. Following the quenching of formaldehyde/paraformaldehyde with 100 mM Tris-HCl pH 7.5, the cells were permeabilized with 150 mM NaCl, 100 mM Tris-Cl pH 7.5, 2 mM MgCl_2_, 0.2% Triton X-100, 0.5% fish skin gelatin (FSG), and 0.5 mM PMSF 0.2% Triton X-100 for 12 min. Probing with the primary (anti-SRPK1 monoclonal antibody diluted 1:150, BD Biosciences, San Jose, CA, USA; anti-SRPK2 monoclonal antibody diluted 1:200, BD Biosciences; anti-FLAG monoclonal antibody diluted 1:1500, Sigma-Aldrich; anti-H2AX diluted 1:200) and secondary (FITC-conjugated goat anti-mouse diluted 1:400, Molecular Probes, Eugene, OR, USA) antibodies and DNA staining (propidium iodide) were performed as previously described [6]. The anti-phospho-histone H2AX (Ser139), clone JBW301, monoclonal antibody (Millipore, Billerica, MA, USA) was kindly provided by D. Thanos (Biomedical Research Foundation, Athens, Greece). After three washes, the coverslips were mounted in 90% glycerol and visualized in a Nikon confocal microscope using the EZ-C1 3.20 software (Nikon Inc., Melville, NY, USA). All chemicals were purchased from Sigma-Aldrich, Darmstadt, Germany.

### 2.4. MTT Assays—Optical Microscopy

The HeLa and T24 cells were seeded in 96-well plates (3 × 10^4^ HeLa or 5 × 10^4^ T24 cells per well) and after 24 h, they were exposed to chemotherapeutic agents (5-FU, cisplatin) and kinase inhibitors (SRPIN340, KU-55,933, VE-821), either alone or in combination, as indicated. The viability of the cells was estimated using a 3-(4,5-imethylthiazol-2-yl)-2,5-diphenyltetrazolium bromide (MTT, Sigma-Aldrich, Darmstadt, Germany) metabolic assay, as described previously [28]. The values shown represent the means ± standard errors of three independent experiments run in triplicate. The density and morphology of control and treated cells were also observed using an inverted phase-contrast microscope (Nikon ECLIPSE TS100, Nikon Inc.Melville, NY, USA) at 10× magnification. Images were captured at the same time points as the MTT and immunofluorescence assays.

### 2.5. Cell Fractionation, SDS-PAGE, and Western Blotting

The REAP (Rapid Efficient And Practical) method [29] was used for the subcellular fractionation of HeLa and T24 cells. Cells grown at a confluency of 60–70% in 10 cm diameter dishes were washed in ice-cold phosphate buffer saline (PBS), scraped from culture dishes on ice using a plastic cell lifter, and harvested in 1 mL PBS. After centrifugation for 10 s in an Eppendorf tabletop microfuge (Eppendorf AG, Hamburg, Germany), supernatants were removed and cell pellets were resuspended in ice-cold PBS containing 0.1% NP40 (control cells were resuspended in 500 μL, cells treated with 5-FU or cisplatin in 300 μL) and homogenized 5 times using a p1000 Gilson micropipette (Gilson, Dunstable, UK). One-third of the lysate was removed as the “whole-cell extract,” the protein concentration was determined using the Bradford assay, and then a 5 × Laemmli sample buffer was added to it and kept on ice. The remaining two-thirds were centrifuged for 10 s, the supernatant was removed as the “cytosolic fraction,” a 5 × Laemmli sample buffer was added to it, and then it was boiled for 2 min. The pellet was resuspended in 1 mL of ice-cold PBS with 0.1% NP40, centrifuged for 10 s, the supernatant was discarded, and the new pellet was resuspended in a 1 × Laemmli sample buffer and designated as the “nuclear fraction” (nuclear fractions from control cells were resuspended in 100 μL, while nuclear fractions from cells treated with 5-FU or cisplatin in 60 μL). Whole extracts and nuclear fractions were passed (≈10 times) through a 25-gauge syringe needle and then boiled for 2 min. Gel loading was adjusted to give equivalent cell numbers in each lane and samples were analyzed on 10% SDS-PAGE. SRPK1 and SRPK2 were detected via Western blotting using the respective anti-SRPK1 and anti-SRPK2 monoclonal antibodies, an alkaline phosphatase-coupled goat anti-mouse secondary antibody, and a 5-bromo-4-chloro-3-indolyl phosphate/nitro blue tetrazolium substrate. To test the purity of cytosolic and nuclear fractions, a mouse monoclonal α-GAPDH (kindly provided by A. Bakopoulou, School of Dentistry, Aristotle University, Thessaloniki, Greece) and a polyclonal anti-lamin (α-L1) (kindly provided by S. Georgatos, Medical School, University of Ioannina, Ioannina, Greece) were used.

Transfected HeLa cells were lysed in 200 μL of 1% Triton buffer (1% Triton X-100, 50 mM Tris-HCl pH 7.5, 150 mM NaCl, and 1 mM PMSF). Whole-cell extracts were clarified via centrifugation for 15 min at 13,000× *g* in a microcentrifuge and analyzed on 10% SDS-PAGE. Gel loading was adjusted to give equivalent cell numbers in each lane and Western blotting was performed with the M5 anti-FLAG monoclonal antibody (Sigma). Coomassie Brilliant Blue (CBB) staining of bacterially produced recombinant proteins was performed according to standard procedures. All chemicals were purchased from Sigma-Aldrich, Darmstadt, Germany.

### 2.6. In Vitro Kinase Assays

Kinase assays were carried out in a total volume of 25 μL containing GST-SRPK1 or GST-SRPK1 mutants (1 μg each) as the substrate with 25 μM ATP, 1 μCi of [γ-^32^P]ATP and 0.1 μg activated Akt or 42 units (0.5 μL) DNA-PK for 30 min at 30 °C. Recombinant active Akt1 and DNA-PK were purchased from Upstate Biotechnology (Catalog#14–276, current supplier Millipore, Billerica, MA, USA) and Promega, Madison, WI, USA, respectively. The reaction buffer for the Akt assays contained 12 mM Hepes pH 7.5 and 10 mM MgCl_2_, while DNA-PK was used as a kinase source, where the assay buffer contained 50 mM Hepes pH 7.5, 50 mM KCl, 10 mM MgCl_2_, 0.2 mM EGTA, 0.1 mM EDTA, 1 mM DTT, and 80 μg/mL BSA. Double-stranded DNA was omitted in the DNA-PK assay since it did not enhance the SRPK1 phosphorylation. A similar DNA-independent mode of action has previously been reported for the phosphorylation of adenoviral L4–33K protein by DNA-PK [30]. To test the activity of SRPK1 mutants similar to Akt kinase, the assays were performed containing GST-SRPK1/GST-SRPK1 mutants (0.5 μg each) and 2 μg GST-LBRNt(62–92) as a substrate. Phosphoproteins were detected via autoradiography using Super RX (Fuji medical X-ray film, Fujifilm, Düsseldorf Germany), and signals were quantified by excising the radioactive bands from the gel and scintillation counting. All chemicals were purchased from Sigma-Aldrich, Darmstadt, Germany.

## 3. Results

### 3.1. DNA Damage Inducers Trigger the Nuclear Translocation of SRPK1

**Figure 1:**
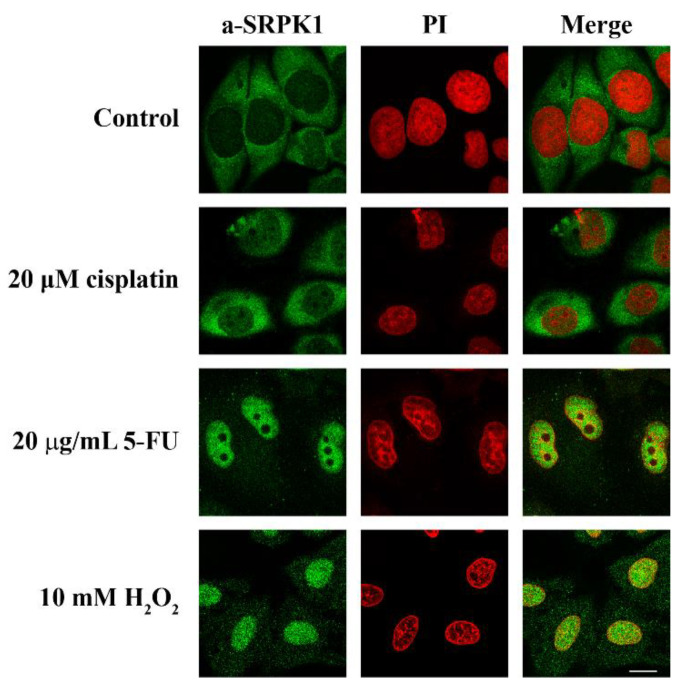
Nuclear translocation of SRPK1 in response to stress agents. HeLa cells were treated with 20 µM cisplatin for 24 h or 20 µg/mL 5-FU for 48 h or 10 mM H_2_O_2_ for 1 h and stained for SRPK1 using the anti-SRPK1 monoclonal antibody. Nuclei were stained with propidium iodide (PI). Scale bar: 10 µM.

Previous reports have demonstrated the induction of SRPKs’ nuclear translocation in response to cellular stress [6,9,13,22]. To further characterize the effectiveness and possibly find a common denominator of the various stress signals, we designed a series of immunofluorescence experiments to test the outcome of stress agents on the SRPK1 redistribution from the cytoplasm to the nucleus, focusing mainly on two widely used chemotherapeutic drugs, namely, 5-FU and cisplatin. In line with previous observations [6,22,23], the nuclear levels of SRPK1 were somewhat increased by 20 μM cisplatin, while the redistribution was far more pronounced upon treatment of HeLa cells with 5-FU (20 μg/mL) and an inducer of oxidative stress, namely, H_2_O_2_ (10 mM), which resulted in almost complete nuclear translocation of SRPK1 (Figure 1).

**Figure 2:**
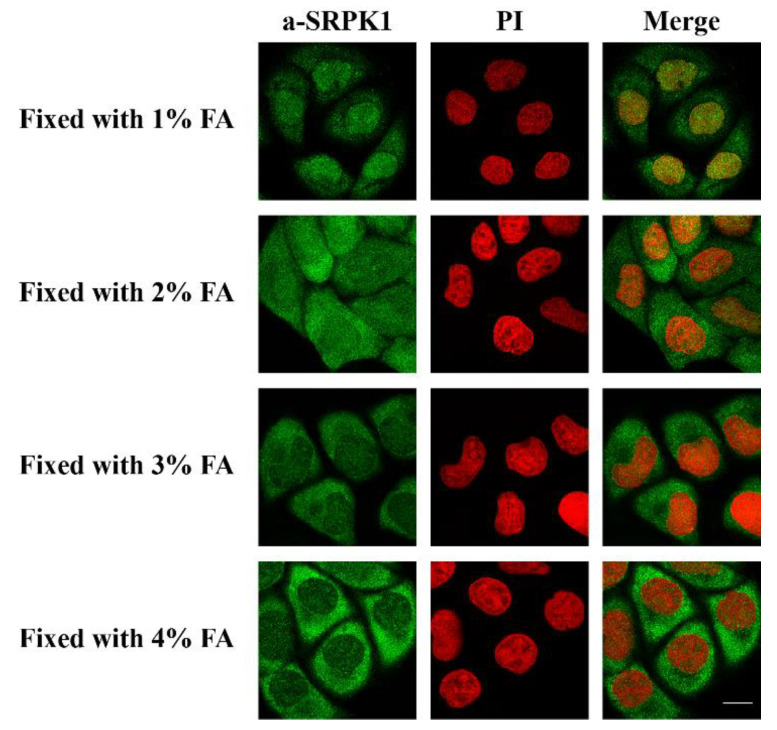
Fixation with various concentrations of formaldehyde (FA) affects the subcellular distribution of SRPK1. Fluorescent patterns of SRPK1 in HeLa cells fixed for 5 min with 1%, 2%, 3%, or 4% FA. SRPK1 was detected using the anti-SRPK1 monoclonal antibody, while nuclei were stained with PI. Scale bar: 10 µM.

Furthermore, while it is clear that SRPK1 exhibits a predominant cytoplasmic localization, in our initial immunofluorescence studies we had detected it mostly in the nucleus [31]. In those experiments, K562 and HeLa cells were fixed with 1% formaldehyde for 5 min, based on a fixation protocol that had been previously used for lamin B receptor (LBR) [32]. Similarly, Wang et al. detected SRPK1 and SRPK2 in both the nucleus and the cytoplasm of HeLa cells fixed with 2% formaldehyde for 10 min [33]. Thus, it appears that fixation conditions might affect the subcellular distribution of SRPKs. In this respect, we checked the localization of SRPK1 following the fixation of HeLa cells with increasing concentrations of formaldehyde for 5 min. As shown in Figure 2, raising the concentration of formaldehyde induced the relocalization of SRPK1 from the nucleus to the cytoplasm. Even though fixation may have an effect on the epitope recognized by the antibody, we also considered the intriguing possibility that fixation with low concentrations of formaldehyde and for short periods, while being efficient for large proteins confined in an intracellular membrane, such as LBR, may be inefficient for mobile proteins that shuttle between the cytoplasm and the nucleus, such as SRPK1. In such a case, formaldehyde may be regarded as a form of stress that induces a rapid nuclear translocation of SRPK1.

**Figure 3:**
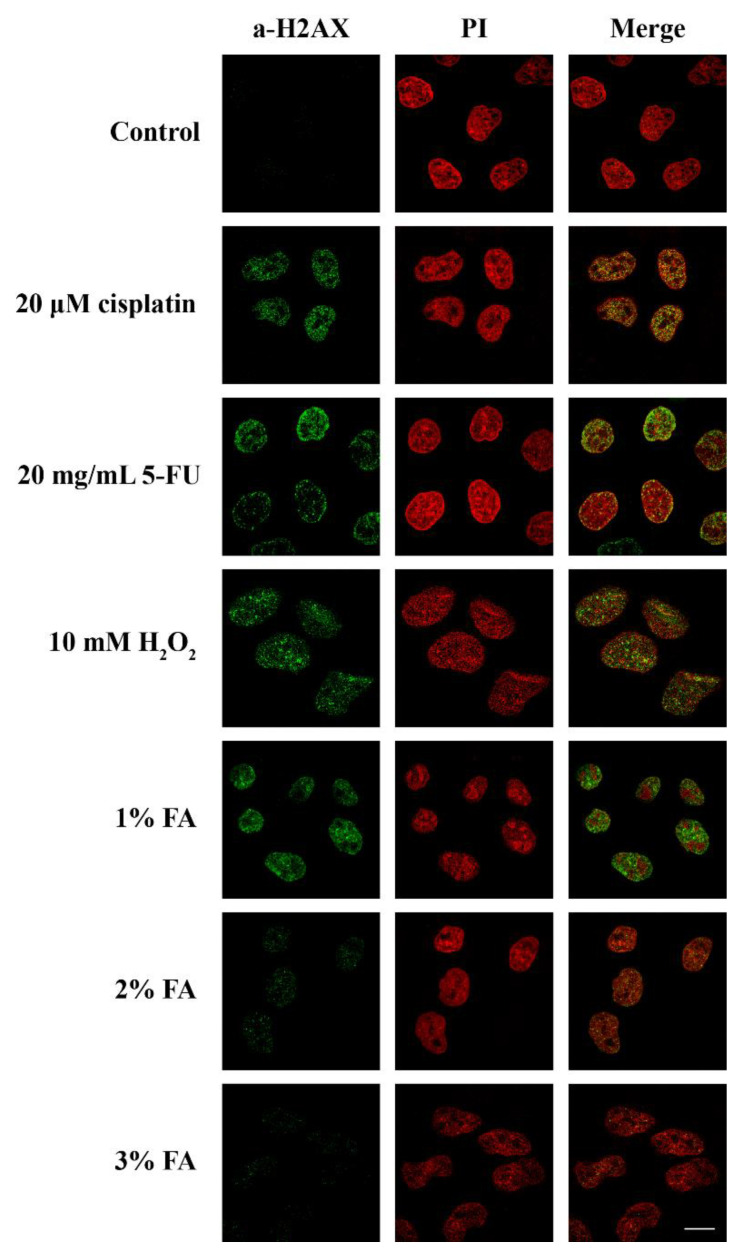
Stress agents and inefficient fixation induced DNA damage. Fluorescent patterns of H2AX in HeLa cells treated with 20 µM cisplatin for 24 h, 20 µg/mL 5-FU for 48 h, 10 mM H_2_O_2_ for 1 h, or fixed for 5 min with 1%, 2%, or 3% FA. H2AX was detected using the anti-phospho-histone H2AX (Ser139) monoclonal antibody (Millipore), while nuclei were stained with PI. Scale bar: 10 µM.

As the most significant consequence of stress in eukaryotic cells is thought to be DNA damage [34], we then checked whether the translocation of SRPK1 to the nucleus might be related to the cellular response to DNA damage. In this respect, we determined the phosphorylation of H2AX, which is a critical event in the DNA damage response, with specificity for double-strand breaks. The phosphorylated form, namely, γH2AX, accumulates along long domains of chromatin that are adjusted to the site of the double-strand break and triggers the recruitment of repair factors [35]. The appearance of γH2AX foci upon various treatments of HeLa cells clearly correlated with the nuclear translocation of SRPK1, with the exception of cisplatin (Figure 1 and Figure 3). Treatment with cisplatin led to an increased number of γH2AX foci, while the kinase partially relocalized to the nucleus. Interestingly, the induction of γH2AX foci, when untreated HeLa cells were fixed with 1% formaldehyde for 5 min, was more or less similar to the one observed following treatment of the cells with 5-FU and H_2_O_2_. The progressive loss of γH2AX foci formation following the fixation of cells with higher concentrations of formaldehyde paralleled the relocalization of SRPK1 from the nucleus to the cytoplasm (Figure 2 and Figure 3), thus confirming the hypothesis that low concentrations of formaldehyde, even for the short period of fixation, may indeed function as a genotoxic agent, resulting in DNA double-strand breaks and SRPK1 nuclear translocation.

### 3.2. SRPIN340 Protected HeLa and T24 Cells from the Cytotoxic Effects of 5-FU and Cisplatin

**Figure 4:**
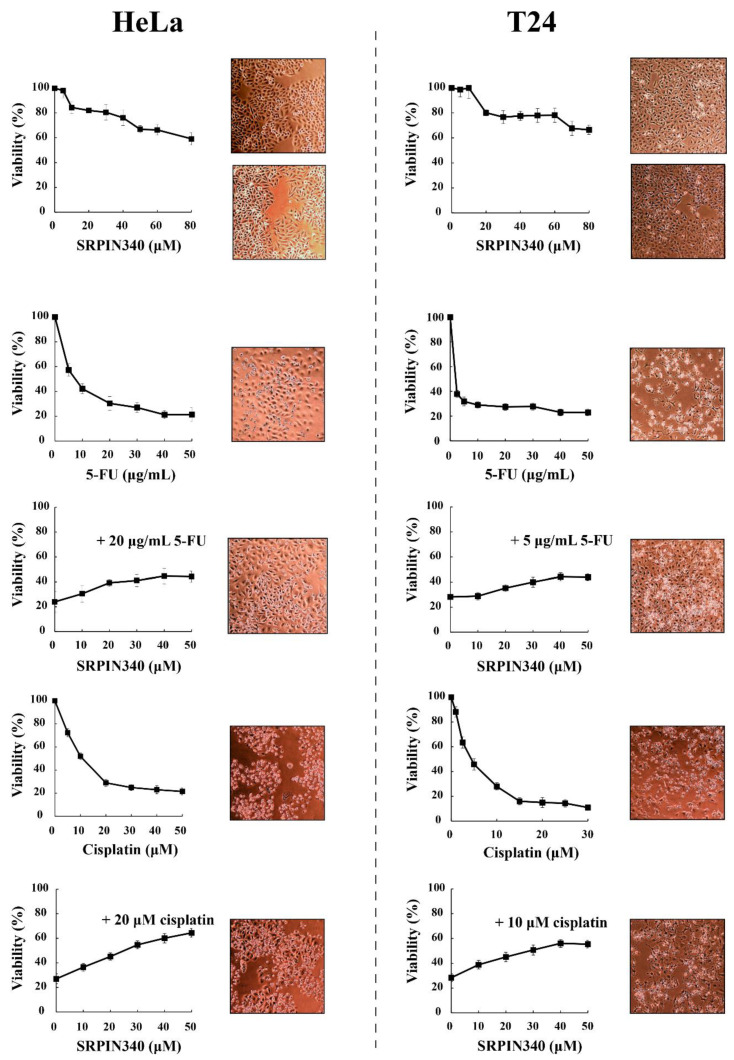
SRPIN340 protected HeLa and T24 cells from the cytotoxic effects of 5-FU and cisplatin. HeLa cells (**left panel**) were treated with SRPIN340 (5–80 µM) for 48 h, 5-FU (5–50 μg/mL) for 48 h, 5-FU (20 μg/mL) plus SRPIN340 for 48 h, cisplatin (5–50 µM) for 24 h, or cisplatin (20 µM) plus SRPIN340 for 24 h. T24 cells (**right panel**) were treated with SRPIN340 (5–80 µM) for 48 h, 5-FU (2.5–50 μg/mL) for 48 h, 5-FU (5 μg/mL) plus SRPIN340 for 48 h, cisplatin (1–30 µM) for 48 h, or cisplatin (10 µM) plus SRPIN340 for 48 h. The number of viable cells was measured using an MTT assay. Viability is expressed as a percentage of the viability of untreated cells, which was set to 100 percent. The density and morphology of control and treated cells were also observed using an inverted phase-contrast microscope at 10× magnification. Images were captured at the same time points as the respective MTT assays.

A key question arising from the above data is whether nuclear translocation and the activity of SRPK1 is necessary to produce the cytotoxic effect or, on the contrary, is part of the signaling pathways involved in the repair mechanism and/or promoting cell resistance to genotoxic agents. To address this issue and to further probe into the partial SRPK1 responsiveness upon cisplatin treatment, we co-treated HeLa and T24 cells with 5-FU or cisplatin and SRPIN340, a specific inhibitor of SRPKs [36], and examined the viability of the cells in parallel with the localization of SRPK1. As shown in Figure 4, the treatment of HeLa and T24 cells with various concentrations of SRPIN340 for 48 h had a rather limited effect on the cell viability (≈60% cell viability at a concentration of 80 μM). SRPIN340 did not exhibit a combinatorial effect with the chemotherapeutic drugs; on the contrary, it conferred a 2–3-fold resistance to 5-FU and cisplatin. This observation was further substantiated using inverted phase-contrast microscopy, which highlighted the higher number and better morphology of cells co-treated with SRPIN340 and 5-FU or cisplatin (Figure 4). Interestingly, we noticed that the most potent protective effects of SRPIN340 were achieved at drug concentrations resulting in 20–25% cell viability. Based on this observation, HeLa cells were treated in the combinatorial assays with 20 μg/mL 5-FU for 48 h and 20 μM cisplatin for 24 h (HeLa cells were more sensitive to cisplatin and, therefore, the time of treatment was reduced), while T24 cells were treated for 48 h with 5 μg/mL 5-FU and 10 μM cisplatin.

### 3.3. SRPIN340 Prevented the Nuclear Translocation of SRPK1 and SRPK2 in 5-FU- and Cisplatin-Treated Cells

**Figure 5:**
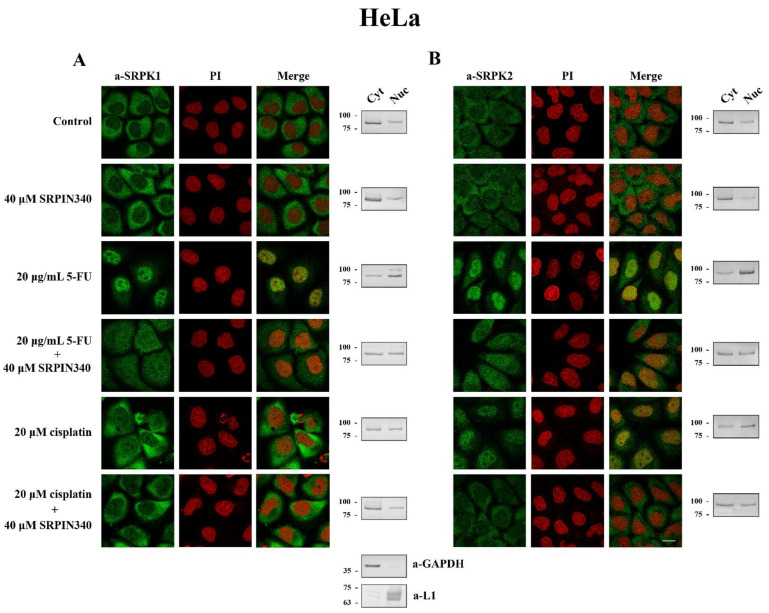
SRPIN340 prevented the nuclear translocation of SRPK1 and SRPK2 in 5-FU- and cisplatin-treated HeLa cells. Fluorescent patterns of SRPK1 ((**A**), **left panel**) and SRPK2 ((**B**), **left panel**) in HeLa cells treated with 40 µM SRPIN340 for 48 h, 20 µg/mL 5-FU for 48 h, 20 µg/mL 5-FU plus 40 µM SRPIN340 for 48 h, 20 µM cisplatin for 24 h, or 20 µM cisplatin plus 40 µM SRPIN340 for 24 h. SRPK1 and SRPK2 were detected using the respective anti-SRPK1 and anti-SRPK2 monoclonal antibodies, while the nuclei were stained with PI. Scale bar: 10 µM. A cell fractionation method (described in the Materials and Methods section) was also employed to analyze the subcellular distribution of SRPK1 ((**A**), **right panel**) and SRPK2 ((**B**), **right panel**). Cytoplasmic (Cyt) and nuclear (Nuc) extracts were subjected to Western blotting using the respective anti-SRPK1 and anti-SRPK2 monoclonal antibodies. The distribution of GAPDH (a-GAPDH) and lamins (a-L1) was used as a marker to assess the fractionation efficiency.

**Figure 6:**
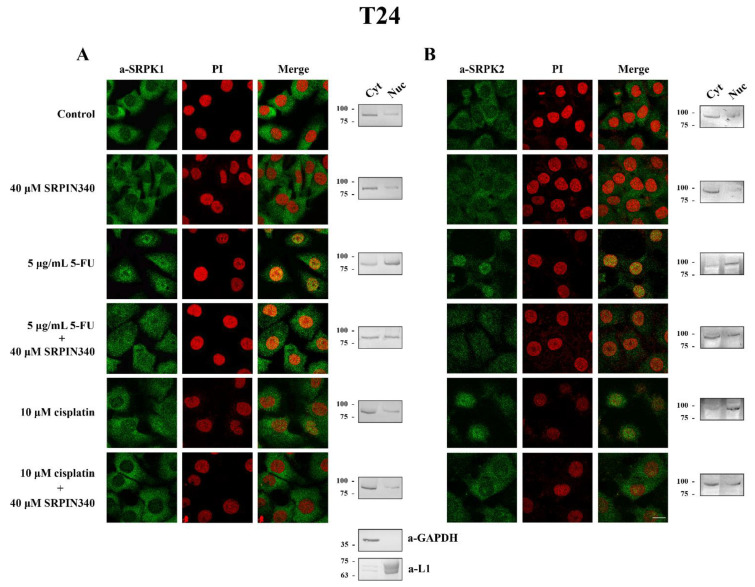
SRPIN340 prevented the nuclear translocation of SRPK1 and SRPK2 in 5-FU- and cisplatin-treated T24 cells. Fluorescent patterns of SRPK1 ((**A**), **left panel**) and SRPK2 ((**B**), **left panel**) in T24 cells were treated with 40 µM SRPIN340 for 48 h, 5 µg/mL 5-FU for 48 h, 5 µg/mL 5-FU plus 40 µM SRPIN340 for 48 h, 10 µM cisplatin for 48 h, or 10 µM cisplatin plus 40 µM SRPIN340 for 48 h. SRPK1 and SRPK2 were detected using the respective anti-SRPK1 and anti-SRPK2 monoclonal antibodies, while the nuclei were stained with PI. Scale bar: 10 µM. A cell fractionation method (described in the Materials and Methods section) was also employed to analyze the subcellular distribution of SRPK1 ((**A**), **right panel**) and SRPK2 ((**B**), **right panel**). Cytoplasmic (Cyt) and nuclear (Nuc) extracts were subjected to Western blotting using the respective anti-SRPK1 and anti-SRPK2 monoclonal antibodies. The distribution of GAPDH (a-GAPDH) and lamins (a-L1) was used as a marker to assess the fractionation efficiency.

While on its own, SRPIN340 did not affect the predominant cytoplasmic localization of SRPK1, it did significantly prevent the nuclear translocation of the kinase in 5-FU-treated HeLa (Figure 5A, left panel) and T24 cells (Figure 6A, left panel), corroborating previous reports that the kinase activity is critical for the entry of SRPK1 into the nucleus [6,8]. A similar effect of SRPIN340 was also observed in cisplatin-treated cells, even though SRPK1 relocalized to the nucleus to a lesser extent (Figure 5A, left panel; Figure 6A, left panel). Interestingly, we noticed that despite the significantly more pronounced nuclear translocation of SRPK1 in 5-FU-treated cells than in cisplatin-treated cells, co-treatment with SRPIN340 protected the cells from both drugs; furthermore, the observed resistance was somewhat higher in cisplatin-treated cells than in 5-FU-treated cells. Considering that SRPIN340 inactivates both SRPK1 and SRPK2 [36], as well as previous reports in the literature showing that SRPK2 relocalized in the nucleus upon cisplatin treatment [13,22], we next examined the subcellular distribution of SRPK2 following 5-FU or cisplatin treatment. As shown in Figure 5B’s left panel (HeLa cells) and Figure 6B’s left panel (T24 cells), both 5-FU and cisplatin caused an almost complete nuclear translocation of SRPK2, while co-treatment with SRPIN340 hindered the nuclear accumulation of the kinase. Cellular fractionation experiments confirmed the immunofluorescence data (Figure 5 and Figure 6, right panels). Taken together, the above findings indicate that the kinase nuclear translocation and activity were essential to the involvement of SRPKs in the cytotoxicity of 5-FU and cisplatin.

### 3.4. Phosphorylation of Thr326 and Ser408 Was Necessary but Not Sufficient for the Nuclear Translocation of SRPK1

We next wished to probe the mechanism driving the nuclear import of SRPK1 after treatment with genotoxic agents. Taking into account the fact that the nuclear translocation of the kinase occurs rapidly and phosphorylation has already been implicated in this process [11], we attempted to identify potential phosphorylation sites. According to Scansite 4.0 (http://scansite4.mit.edu), which is a motif–profile scoring algorithm that takes into consideration not only the phosphorylation motif but also the influence of the neighboring residues [37], a threonine (Thr326) and two serine (Ser51, Ser408) residues were considered high-stringency hits. We had previously shown that Ser51, which is located within the first catalytic domain, was phosphorylated in vitro by casein kinase 2 (CK2), resulting in partial activation of SRPK1 [26]. Accordingly, we determined whether phosphorylation of this site had any effect on the nuclear translocation of SRPK1. Yet, the phosphorylation-defective mutant, SRPK151A, quantitatively shifted to the nucleus upon treatment of the HeLa cells with 40 μg/mL 5-FU for 48 h (Figure S1), suggesting that the modification of Ser51 was not responsible for the nuclear accumulation of the kinase. The other two predicted sites (Thr326 and Ser408) were more likely to be involved since they were both located within the spacer region that mediates the cytoplasmic sequestration of SRPK1 [9]. In addition, Thr326 was one of the two sites that were reported to be phosphorylated by activated Akt following EGF signaling [11].

**Figure 7:**
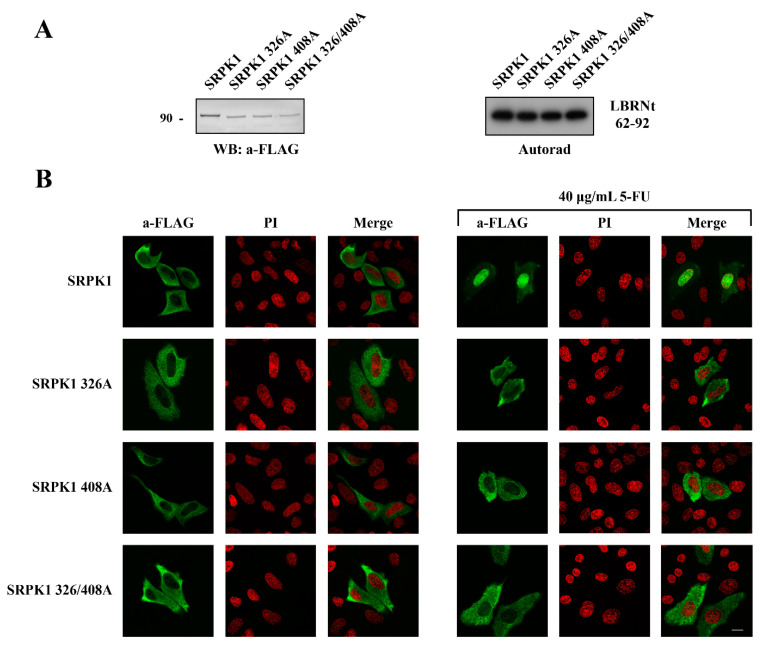
Phosphorylation of Thr326 and Ser408 was required for the nuclear translocation of SRPK1. (**A**) Western blot of the cell extracts prepared from HeLa cells transfected with FLAG-SRPK1, FLAG-SRPK1326A, FLAG-SRPK1408A, and FLAG-SRPK1326/408A (**left panel**). Phosphorylation of GST-LBRNt(62–92) by 0.5 µg wild-type GST-SRPK1, GST-SRPK1326A, GST-SRPK1408A, and GST-SRPK1326/408A (**right panel**). (**B**) Fluorescent pattern of wild-type FLAG-SRPK1, FLAG-SRPK1326A, FLAG-SRPK1408A, and FLAG-SRPK1326/408A in the control and 5-FU-treated HeLa cells. In the transfected cells, the concentration of 5-FU was raised to 40 μg/mL to achieve complete nuclear translocation of FLAG-SRPK1. SRPK1 was detected using the M5 anti-FLAG monoclonal antibody, while nuclei were stained with PI. Scale bar: 10 µM.

Therefore, we mutated both Thr326 and Ser408 to alanine, either individually or in combination, finding that a mutation of either of these two residues prevented the relocation of the kinase from the cytoplasm to the nucleus in response to 5-FU treatment (Figure 7B). Neither the single nor the double mutation had any effect on the kinase activity toward GST-LBRNt(62–92), a well-known substrate of SRPK1 (Figure 7A), ruling out the possibility that restriction of the kinase to the cytoplasm was due to its inactivation.

**Figure 8:**
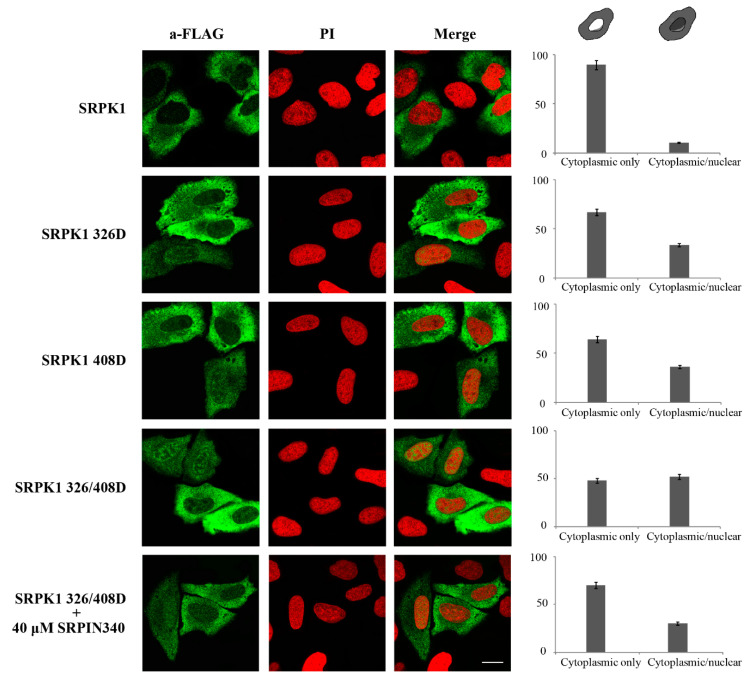
The phosphorylation-mimicking mutants of SRPK1 were partially localized in the nucleus. Representative confocal images of wild-type FLAG-SRPK1, FLAG-SRPK1326D, FLAG-SRPK1408D, FLAG-SRPK1326/408D, and FLAG-SRPK1326/408D plus SRPIN340. SRPK1 was detected using the M5 anti-FLAG monoclonal antibody, while the nuclei were stained with PI. Scale bar: 10 µM. A diagrammatic representation of wild-type FLAG-SRPK1 and mutant FLAG-SRPK1 staining patterns is shown in the upper part of the right panel. The percentage of SRPK1 staining patterns relative to the indicated staining pattern was determined for ≈150 cells in two different experiments, where the means ± standard errors of the measurements are shown.

This evidence indicates that both these sites appeared to be induced by genotoxic agents; thus, both were critical for the nuclear translocation of SRPK1. As the effect of phosphorylation can be imitated by introducing negatively charged residues, we substituted Thr326 and Ser408 with aspartic acid. While the phosphorylation-defective mutants (SRPK1326A, SRPK1408A, SRPK1326/408A) were almost completely restricted to the cytoplasm, the phosphorylation-mimicking mutants (SRPK1326D, SRPK1408D, SRPK1326/408D) were partially localized in the nucleus in the absence of 5-FU treatment, with the relocation being more notable for the double mutant (Figure 8). In accordance with our previous data, the treatment of SRPK1326/408D-transfected cells with SRPIN340 significantly increased the percentage of cells that exhibited prominent cytoplasmic localization.

Although Asp is singly charged, whereas pThr and pSer are doubly charged at physiological pH, and therefore, the partial nuclear translocation may be due to reduced negative charge of the double phosphomimetic mutant as compared to phosphorylated SRPK1, these data may also imply that the phosphorylation of Thr326 and Ser408 was necessary but not sufficient for the nuclear translocation of SRPK1 and an additional modification(s) is (are) required.

### 3.5. ATR/ATM-Dependent Phosphorylation of Thr326 and Ser408

In a subsequent step, we sought to identify the kinase responsible for targeting Thr326 and Ser408. Since activated Akt has previously been reported to induce SRPK1 autophosphorylation on Thr326 [11], we first tested whether recombinant active Akt1 could induce the phosphorylation of these two sites. Akt1 phosphorylated or induced phosphorylation of bacterially expressed SRPK1 (GST-SRPK1) with very low stoichiometry (<0.03 moles ATP/mole GST-SRPK1) (Figure S2A), while we did not observe any differences between wild-type and mutant SRPK1.

Thr326 and Ser408 are within the S/TQ motifs that are preferred phosphorylation sites for PI3K-like kinases. According to Scansite 4.0, the predicted kinase on these sites was DNA-PK, which belongs to the PI3K-like family of serine/threonine protein kinases and plays an essential role in DNA damage signaling [38]. However, recombinant DNA-PK also phosphorylated bacterially expressed SRPK1 (GST-SRPK1) with very low stoichiometry (≈0.05 moles ATP/mole GST-SRPK1), while, even at this low stoichiometry, DNA-PK phosphorylated wild-type and mutant GST-SRPK1s with the same efficiency (Figure S2B). To further confirm that DNA-PK was not the responsible kinase, we repeated the immunofluorescence assays using M059K cells, which express normal levels of DNA-PK, and M059J cells, which lack DNA-PK activity. As shown in Figure S2C, in both cell lines, treatment with 5-FU resulted in almost complete nuclear translocation of SRPK1.

**Figure 9:**
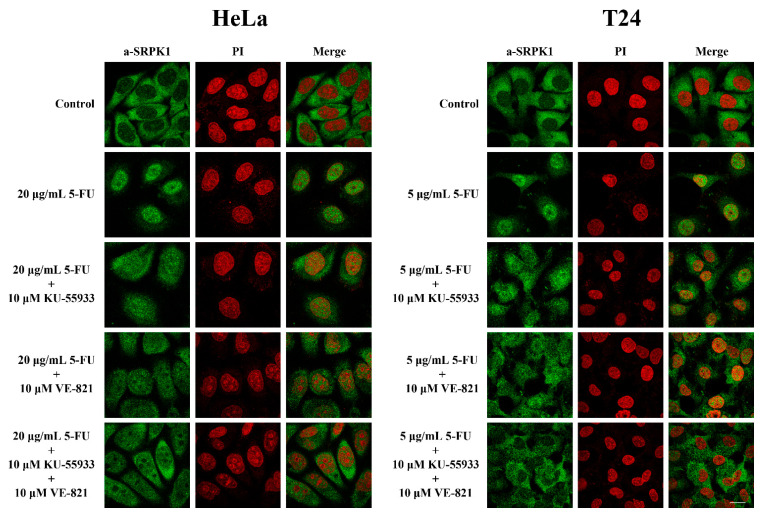
ATR/ATM inhibition significantly prevented the nuclear translocation of SRPK1 in 5-FU-treated HeLa and T24 cells. Fluorescent patterns of SRPK1 in HeLa (**left panel**) and T24 cells (**right panel**) treated either alone with 5-FU (20 µg/mL in HeLa, 5 µg/mL in T24) for 48 h, or co-treated for 48 h with 5-FU plus 10 µM KU-55,933, 5-FU plus 10 µM VE-821, or 5-FU plus 10 µM KU-55,933 plus 10 µM VE-821. SRPK1 was detected using the anti-SRPK1 monoclonal antibody, while nuclei were stained with PI. Scale bar: 10 µM.

Taking into consideration the fact that Thr326 and Ser408 are within a consensus targeted by PI3K-like kinases and DNA-PK was not the upstream kinase, we next sought to investigate whether ATM and/or ATR, which are the two other main members of the PI3K-like family, were responsible for phosphorylating these two residues. In this respect, PhosphoNET, another predictor of human phosphorylation sites, hints toward ATR as being the main candidate kinase for targeting Thr326 and Ser408. As shown in Figure 9, mainly the inhibition of ATR, or ATM to a lesser extent, by VE-821 and KU-55,933, respectively, led to reduced nuclear localization of SRPK1 in 5-FU-treated cells. Even more interestingly, dual inhibition of ATR and ATM significantly prevented the nuclear accumulation of SRPK1.

**Figure 10:**
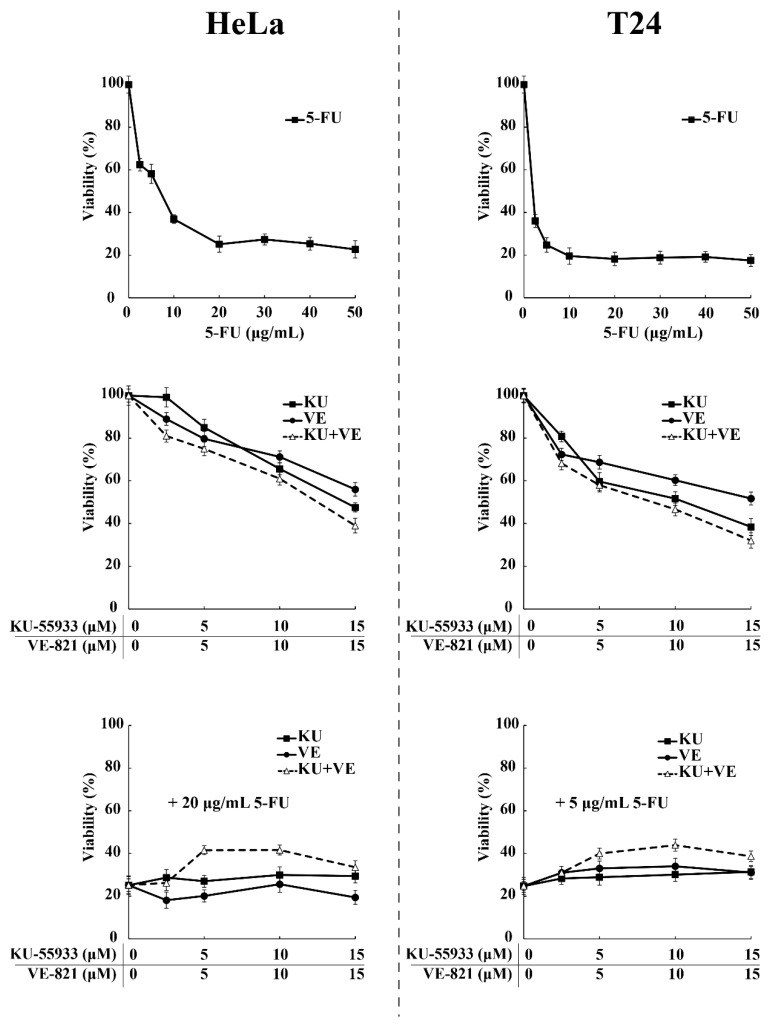
ATR/ATM inhibition protects HeLa and T24 cells from the cytotoxic effects of 5-FU. HeLa (**left panel**) and T24 cells (**right panel**) were treated for 48 h with increasing concentrations of 5-FU and ATR/ATM inhibitors, either alone or in combination, as indicated. The number of viable cells was measured using an MTT assay. Viability is expressed as a percentage of the viability of untreated cells, which was set to 100 percent.

According to our previous data, preventing the nuclear translocation of SRPK1 via pharmacological inhibition of ATR/ATM should exert a beneficial effect on 5-FU-treated cells. To address this issue, we tested the viability of HeLa and T24 cells upon treatment with 5-FU or ATR/ATM inhibitors alone, or in combination (Figure 10). The treatment of cells with increasing concentrations of KU-55,933 or VE-821 or KU-55,933/VE-821 reduced the cell viability at a percentage ranging from ≈40% (VE-821 alone) to ≈60% (KU-55933 plus VE-821). However, dual inhibition of ATR and ATM (KU-55,933/VE-821) did not exhibit a combinatorial effect with 5-FU but conferred a 2–2.2-fold resistance to the drug. Thus, our findings strongly suggest that SRPK1 is a downstream target of ATR/ATM in the DNA damage response.

## 4. Discussion

How genotoxic agents convey signals to the nucleus of cancer cells to mediate their action is not fully understood. Here, we provide evidence that SRPKs are part of these signaling pathways. The nuclear translocation of SRPKs closely correlates with the appearance of γH2AX foci and mediates 5-FU and cisplatin sensitivity.

SRPKs have been reported to translocate into the nucleus upon the stimulation of cells with growth factors [11] and hormones [12], at the late G2 phase [8,14], and after experiencing stress [6,9,13,22]. In the first case, SRPKs function as signal integrators of growth factors and hormones, thus promoting cell growth and survival; in the second case, they promote cell cycle progression through yet uncharacterized mechanisms; in the third case, the functional consequences of the nuclear accumulation of SRPKs induced by stress signals remain rather unclear. In sorbitol-stressed HeLa cells, the entry of SRPK1 into the nucleus induced the hyperphosphorylation of SR proteins, thus becoming inhibitory to the splicing of a reporter gene [9], while genotoxic treatments of human neuroblastoma cells resulted in the nuclear localization of SRPK2 and promoted changes in the splicing pattern of genes involved in DNA repair, cell cycle control, and apoptosis [13]. However, in neither of these two reports is it clarified whether the observed alterations to the RNA splicing landscape mediate the cytotoxic effects of stress agents or, in contrast, protect cells against stress. On the other hand, two other reports provide evidence that the nuclear translocation of SRPK2 is directly related to apoptosis. Edmond et al. showed that, following treatment of non-small cell lung cancer cells with cisplatin, nuclear-localized SRPK2 induced apoptosis through regulation of the splicing switch of caspase-8 pre-mRNA [22], while Jang et al. showed that nuclear import of SRPK2 led to cyclin D1 upregulation, cell cycle re-entry, and neuronal apoptosis [39]. Inhibition of growth was also observed in yeast cells by the forced nuclear accumulation of Sky1p (a yeast homolog of human SRPKs) resulting from the deletion of its spacer sequence [10]. Yet, in a recent report, the nuclear localization of SRPK1 was associated with cisplatin resistance of breast cancer cells [23].

SRPIN340 significantly prevented the nuclear translocation of SRPKs in 5-FU and cisplatin-treated cells, thus diminishing the cytotoxic effects of the drug (Figure 4, Figure 5 and Figure 6). The impaired nuclear accumulation of SRPKs by SRPIN340 was anticipated based on previous findings showing that kinase activity is a prerequisite for the nuclear translocation of SRPKs [6,8]. In line with our observations, the inhibition of SRPK1 by SRPIN340 or trifluoromethyl arylamides maintained SRPK1 predominantly in the cytoplasm when melanoma cells were stimulated with EGF [40]. Similarly, the inhibition of SRPK1 and SRPK2 by SRPIN340 protected cardiomyocytes from oxidative stress-induced apoptosis and cell death, even though the subcellular localization of the kinases was not examined in this report [41].

The increased nuclear levels of SRPKs were primarily, if not exclusively, associated with the increased phosphorylation of SR proteins, leading to alterations of the splicing machinery. Even in the report by Jang et al., cyclin D1 upregulation was mediated by increased phosphorylation of SC35 [39]. However, there is increasing evidence of additional nuclear events in which SRPKs may have a decisive role through the phosphorylation of proteins other than SR splicing factors. In response to apoptotic signals, the nuclear envelope undergoes structural changes, including the detachment of chromatin from the inner nuclear membrane [42]. Lamin B receptor (LBR), a key factor tethering peripheral heterochromatin [43], is a well-characterized substrate of SRPK1 [44,45]. LBR assembles onto oligomers that are entrapped in distinct microdomains in the inner nuclear membrane and bind chromatin [46]. In vitro, phosphorylation of the RS domains results in the dissociation of oligomers with a concomitant increase in solubility [47,48]. In line with this finding, it was shown that, due to the inhibition of protein phosphatase 1, highly phosphorylated LBR was not confined to the nuclear envelope and became dispersed throughout the endoplasmic reticulum [49]. A similar dispersion was observed via phosphomimetic mutation of the serine residues within the RS domain. In contrast, the nuclear envelope localization of LBR was maintained upon treatment of cells with SRPIN340 [47,48,49]. In another study investigating the factors and pathways mediating the cellular response to DNA damage, Boeing et al. analyzed the UV-induced phosphoproteome, while in parallel, they performed a genome-wide siRNA screen, assessing gene products that affect transcription after UV-irradiation [24]. Interestingly, SRPK1 was one of the main kinases that scored in the RNAi screen and was also associated with 20 proteins showing UV-induced phosphorylation. Based on these data, the authors proposed a new role for SRPK1 and its partners in the transcription-related DNA damage response.

In line with the analysis performed by Boeing et al. [24], we provide evidence that SRPK1 was actually involved in the DNA damage response and was a downstream target of ATR/ATM. The phosphorylation of Thr326 and Ser408 in an ATR/ATM-dependent manner, triggered by the 5-FU treatment, was necessary for the nuclear translocation of the kinase. Blocking the ATR/ATM kinase activity by specific inhibitors prevented the nuclear accumulation of SRPK1 and, similarly to SRPIN340, partially protected cells from the cytotoxic effects of 5-FU. At first sight, this is an unexpected result since ATM/ATR inhibitors are generally thought to sensitize tumor cells to genotoxic agents [50]. In this respect, we believe that there is a threshold concentration of the drug, above which, the cytotoxic effects cannot be reversed. Our data with SRPIN340 and preliminary results with KU-55,933 and VE-821 suggest that the threshold concentration of 5-FU was the one that results in ≈20% cell viability, just when the viability curve in the MTT assays began to plateau. Another critical issue is the delivery time of the ATM/ATR inhibitors relative to the genotoxic agent in combinatorial assays. We noticed that KU-55,933/VE-821 prevented the nuclear translocation of SRPK1 and exerted their beneficial effects when they were added to the cells concomitantly with 5-FU and for the same period.

Zhou et al. also identified Thr326 as a site that was phosphorylated in an Akt-dependent manner after EGF treatment of HeLa cells [11], even though this residue is within an S/TQ motif that is totally unrelated to the Akt consensus, namely, RXRXXS/T. In our study, Akt1 phosphorylated SRPK1 with very low stoichiometry (Figure S2A), while it did not show any preference for Thr326 nor Ser408. At present, we do not know whether SRPK2 is also a downstream target of ATR/ATM, even though there is evidence supporting this notion. Vivarelli et al. reported that phosphorylation of Ser588, which is a CK2 site, was necessary and sufficient for the nuclear accumulation of SRPK2 in stress-treated neuroblastoma cells; however, they clearly showed that caffeine blocks SRPK2 nuclear translocation [13]. Caffeine does not interfere with CK2 activity and is considered a potent inhibitor of ATM and ATR kinases [51]. Phosphorylation of other residues by various kinases has also been implicated in the nuclear translocation of SRPK2. Jang et al. reported that Akt phosphorylates SRPK2 on Thr492 and induces its nuclear localization [39]. Phosphorylation of this residue is sufficient as Thr492D resides in the nucleus. Lee et al. reported that, in HEK293E cells, S6K1 phosphorylates SRPK2 on Ser494, which then primes Ser497 phosphorylation by CK1 [12]. The non-phosphorylatable mutants were distributed to the cytoplasm.

One intriguing issue arising from our data and the data reported in the literature is why growth factor/hormone-mediated nuclear translocation of SRPKs is beneficial to the cell, while, in contrast, stress-induced nuclear accumulation of SRPKs is associated with toxic effects. In our opinion, the answer relies on the balance between the cytoplasmic and nuclear levels of SRPKs, which is critical for the cell. This balance is probably achieved by how and to what extent SRPKs are post-translationally modified. Growth factors and/or hormones induce the regulated nuclear translocation of a rather limited number of SRPK molecules, which then mediate the corresponding biological responses, such as well-defined alterations in the splicing machinery and/or partial reorganization of chromatin, leading to the necessary transcriptional changes. In this respect, sub-stoichiometric levels of phosphorylation, e.g., Akt-induced SRPK1 phosphorylation, while of low importance in in vitro assays, may be functionally significant in the cellular context. On the other hand, genotoxic/stress signals induce stoichiometric phosphorylation that results in the massive nuclear accumulation of SRPKs. It is also possible that additional post-translational modifications, such as acetylation, are also implicated in the nuclear accumulation of SRPKs. The lack of complete nuclear localization of the double-phosphomimetic mutant (FLAG-SRPK1326/408D, Figure 8) is in favor of such a hypothesis. In this respect, Edmond et al. observed almost complete nuclear localization of SRPK1 and SRPK2 in non-small-cell lung cancer cells that were deprived of the acetyl-transferase Tip60, suggesting that blocking acetylation was sufficient for the entry of both kinases into the nucleus [22]. Yet, after a cisplatin treatment that strongly decreased the Tip60 protein levels, they observed almost complete nuclear accumulation of SRPK2, but a rather limited nuclear translocation of SRPK1, similar to the one observed in the present study. On the other hand, Wang et al. reported a cisplatin-induced increase of Tip60 levels in breast cancer cell lines, accompanied by the complete nuclear exclusion of SRPK1 [23].

Overall, our findings strongly suggest a key role for SRPKs in the DNA damage response, with the kinases being actively involved in the genotoxic agents- induced cell death. Yet, more extensive research is required to fully characterize the modifications and the implicated enzymes that dictate the nuclear translocation of the kinases, as well as to unravel the nuclear functions of SRPKs that mediate the downstream signaling events of genotoxic agents.

## 5. Conclusions

**Figure 11:**
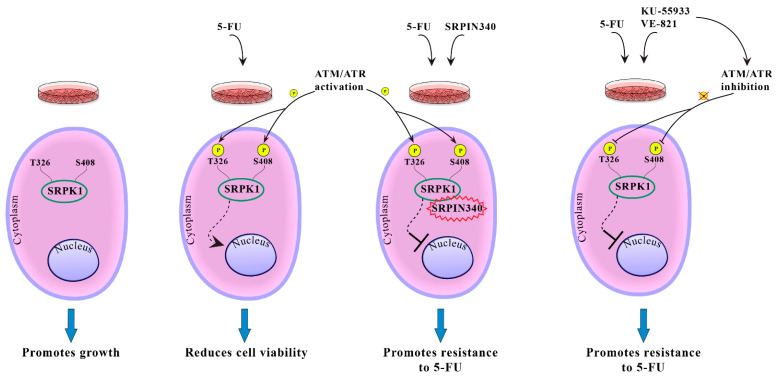
Putative model showing the effect of phosphorylation of Thr326 and Ser408 on the SRPK1 subcellular localization and function.

Summarizing our results, we propose a model for SRPK1 activity and function in relation to its subcellular localization (Figure 11). In untreated cells, SRPK1 localizes almost exclusively in the cytoplasm and promotes cell growth. Treatment with 5-FU induces the phosphorylation of Thr326 and Ser408 in an ATR/ATM-dependent manner, thus resulting in the relocation of the kinase from the cytoplasm to the nucleus. Nuclear SRPK1 mediates the cytotoxic effects of the drug and reduces cell viability. Blocking SRPK1 activity by SRPIN340 or blocking the activity of ATR/ATM by KU-55,933/VE-821 impairs the nuclear translocation of SRPK1 and confers resistance to the drug.

## Supplementary Materials

The following are available online at https://www.mdpi.com/article/10.3390/cells10040759/s1, Figure S1: Phosphorylation of Ser51 is not responsible for the nuclear translocation of SRPK1. Fluorescent pattern of wild-type FLAG-SRPK1 and mutant FLAG-SRPK151A in 5-FU-treated HeLa cells. The concentration of 5-FU was raised to 40 μg/mL to achieve complete nuclear translocation of FLAG-SRPK1. SRPK1 was detected using the M5 anti-FLAG monoclonal antibody, while nuclei were stained with PI. Scale bar, 10 µM; Figure S2: Akt and DNA-PK are not responsible for phosphorylation of Thr326 and Ser408. Phosphorylation of GST-SRPK1, GST-SRPK1326A, GST-SRPK1408A and GST-SRPK1326/408A by recombinant Akt1 (A) and DNA-PK (B). (C) M059K and M059J cells were treated with 20 µg/mL 5-FU for 48 h and stained for SRPK1 using the anti-SRPK1 monoclonal antibody. Nuclei were stained with PI. Scale bar, 10 µM.
