# Supplementary material for: Nuclear Translocation of SRPKs Is Associated with 5-FU and Cisplatin Sensitivity in HeLa and T24 Cells
Source: Cells. 2021 Mar 30;10(4):759. doi: 10.3390/cells10040759 (PMC8065462; doi:10.3390/cells10040759)
Supplement: Supplementary file 1 [file cells-10-00759-s001.pdf]

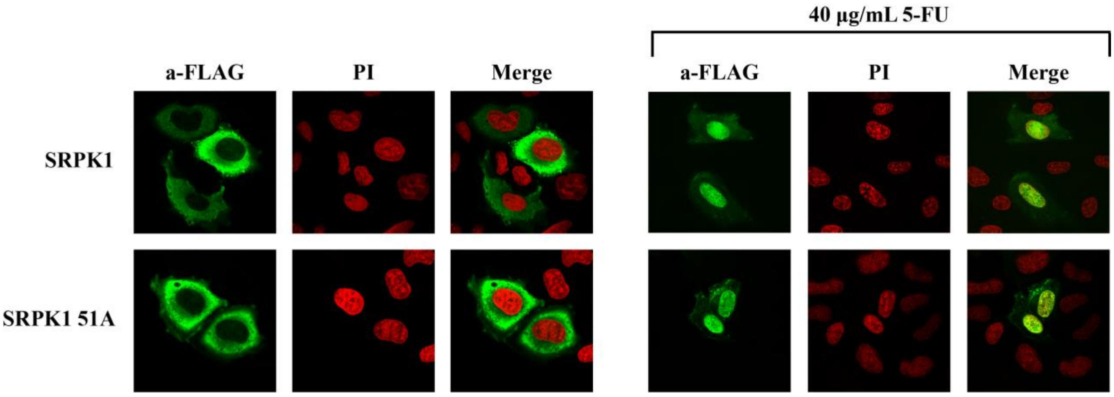

Figure 1. Phosphorylation of Ser51 is not responsible for the nuclear translocation of SRPK1. Fluorescent pattern of wild-type FLAG-SRPK1 and mutant FLAG-SRPK151A in 5-FU-treated HeLa cells. The concentration of 5-FU was raised to 40 μg/mL to achieve complete nuclear translocation of FLAG-SRPK1. SRPK1 was detected using the M5 anti-FLAG monoclonal antibody, while nuclei were stained with PI. Scale bar, 10 μM.

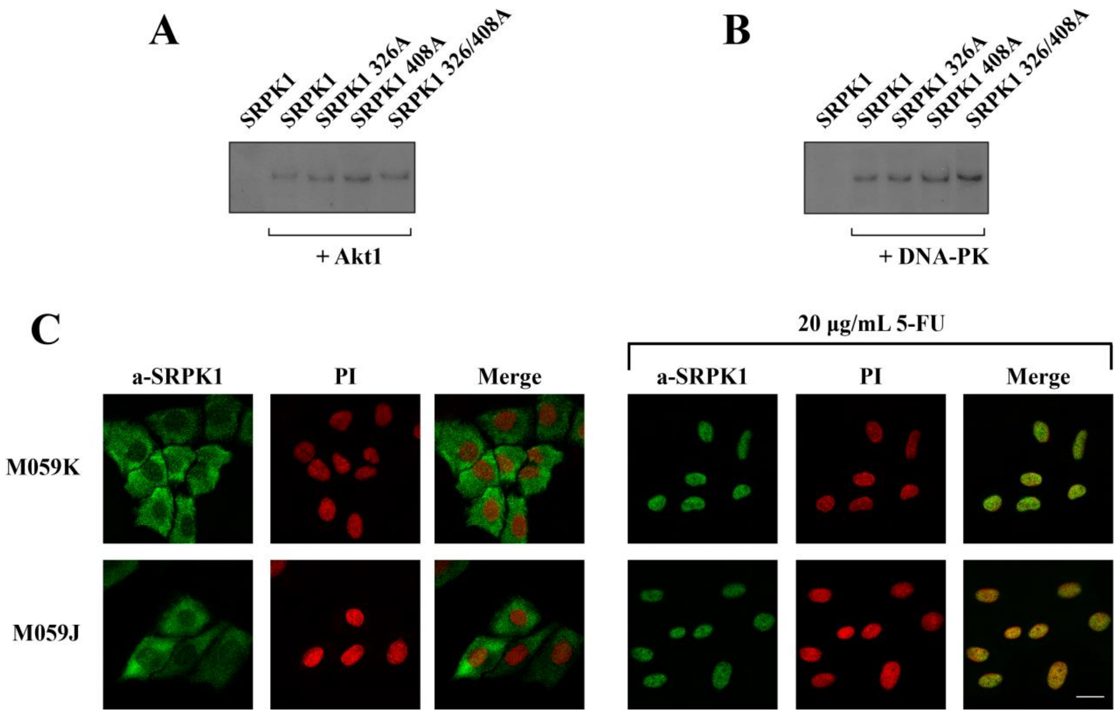

Figure S2. Akt and DNA-PK are not responsible for phosphorylation of Thr326 and Ser408. Phosphorylation of GST-SRPK1, GST-SRPK1326A, GST-SRPK1408A and GST-SRPK1326/408A by recombinant Akt1 **(A)** and DNA-PK **(B)**. **(C)** M059K and M059J cells were treated with 20 µg/mL 5-FU for 48 h and stained for SRPK1 using the anti-SRPK1 monoclonal antibody. Nuclei were stained with PI. Scale bar, 10 µM.
